# Synthesis of the Antimicrobial Peptide Murepavadin Using Novel Coupling Agents

**DOI:** 10.3390/biom14050526

**Published:** 2024-04-27

**Authors:** Júlia García-Gros, Yolanda Cajal, Ana Maria Marqués, Francesc Rabanal

**Affiliations:** 1 Section of Organic Chemistry, Department of Inorganic and Organic Chemistry, Faculty of Chemistry, Universitat de Barcelona, 08028 Barcelona, Spain; juliagarciagros@ub.edu; 2 Department of Pharmacy, Pharmaceutical Technology and Physical Chemistry, Faculty of Pharmacy and Food Sciences, Universitat de Barcelona, 08028 Barcelona, Spain; ycajal@ub.edu; 3 Institute of Nanoscience and Nanotechnology (IN2UB), Universitat de Barcelona, 08028 Barcelona, Spain; 4 Laboratory of Microbiology, Faculty of Pharmacy and Food Sciences, Universitat de Barcelona, 08007 Barcelona, Spain; ammarques@ub.edu

**Keywords:** peptide synthesis, antimicrobial cyclic peptide, murepavadin, acylation agents, solid phase, antibiotic, antibacterial activity

## Abstract

The problem of antimicrobial resistance is becoming a daunting challenge for human society and healthcare systems around the world. Hence, there is a constant need to develop new antibiotics to fight resistant bacteria, among other important social and economic measures. In this regard, murepavadin is a cyclic antibacterial peptide in development. The synthesis of murepavadin was undertaken in order to optimize the preparative protocol and scale-up, in particular, the use of new activation reagents. In our hands, classical approaches using carbodiimide/hydroxybenzotriazole rendered low yields. The use of novel carbodiimide and reagents based on OxymaPure^®^ and Oxy-B is discussed together with the proper use of chromatographic conditions for the adequate characterization of peptide crudes. Higher yields and purities were obtained. Finally, the antimicrobial activity of different synthetic batches was tested in three *Pseudomonas aeruginosa* strains, including highly resistant ones. All murepavadin batches yielded the same highly active MIC values and proved that the chiral integrity of the molecule was preserved throughout the whole synthetic procedure.

## 1. Introduction

Peptides are a class of chemical compounds that offer immense potential as therapeutic tools. These biomolecules are highly selective and effective, as they can bind to specific cell surface receptors and trigger intracellular effects while being relatively safe and well tolerated. Consequently, there is a rising interest in therapeutic peptides, and nowadays, more than eighty peptide drugs have already been approved worldwide [1,2,3]. Since the introduction of solid-phase peptide synthesis (SPPS) by Bruce Merrifield in 1963 [4], this field has experienced a boost in research and development [5]. However, scale-up and production are still complex issues during pharmaceutical development. In fact, small chemical optimization may imply great improvements in yields and purities due to the repetitive nature of the peptide assembly process [3].

In recent decades, a vast arsenal of coupling reagents has been developed for peptide bond formation [6,7,8], including aminium salts of benzotriazoles, such as 2-(1*H*-benzotriazole-1-yl)-1,1,3,3-tetramethyluronium hexafluorophosphate (HBTU) [9]; phosphonium salts, such as (Benzotriazol-1-yloxy)tris(dimethylamino)phosphonium hexafluorophosphate (BOP) or (Benzotriazol-1-yloxy)tripyrrolidinophosphonium hexafluorophosphate (PyBOP) [10], and carbodiimides, among others. Carbodiimides, in fact, are still the compounds of choice because of their lower cost and good performance in non-hindered peptide bond formation in comparison with more complex reagents.

For solution-phase peptide synthesis, *N*-ethyl-*N*′-(3-(dimethylamino)propyl)carbodiimide (EDC) is commonly used because its urea is highly soluble in aqueous solutions. On the other hand, *N*,*N*′-diisopropylcarbodiimide (DIC) is preferred in SPPS because the generated urea byproduct is soluble in organic solvents, while the urea of dicylohexylcarbodiimide (DCC) is prone to precipitation. It is well known, though, that carbodiimides can induce a certain degree of epimerization on the alpha-C of the activated acid group [6]. For this reason, additives, such as 1-hydroxybenzotriazole (HOBt) or 1-hydroxy-7-azabenzotriazole (HOAt) [11], are commonly used. These reagents reduce the high reactivity of O-acylisourea by forming intermediate active esters and inhibit side-reactions, such as racemization, through oxazolones or the formation of *N*-acylureas [12].

**Figure 1:**
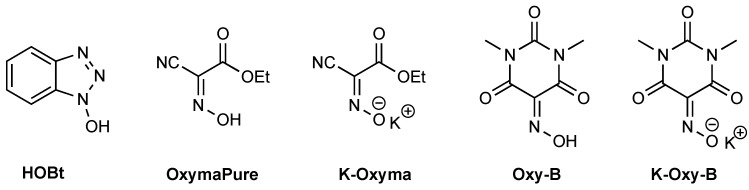
Structures of HOBt and oxyma-based reagents.

Unfortunately, additives, such as HOBt, can induce a certain premature cleavage of the peptide chains, especially when using acid-labile trityl-based resins, because of their acidic nature [13]. Moreover, HOBt and HOAt are classified in the Class I category according to the U.S. Department of Transportation (DOT) and United Nations (UN) guidelines due to their explosive character, and hence, their use is restricted [14]. In this sense, ethylcyano(hydroxyimino)acetate (OxymaPure, first described in 1973 by Itoh [15]) was introduced and was shown to perform better than HOBt in terms of yield and epimerization, and more importantly, it also showed better thermal stability [16]. Later, other oxyma-based compounds were developed, including the COMU reagent ((1-cyano-2-ethoxy-2-oxoethylideneaminooxy)-dimethyl-morpholino-carbeniumhexafluorophosphate [17]) or (Z)-ethyl 2-cyano-3-hydroxyacrylate potassium salt (K-Oxyma) [18], among others (Figure 1). The latter is the potassium salt of OxymaPure, a more appropriate choice when dealing with highly acid-labile resins due to the lack of the acid *N*-hydroxy proton (Figure 1) [18].

However, the combination of DIC with OxymaPure in dimethylformamide (DMF) at 20 °C was demonstrated to lead to the formation of oxadiazole and the harmful volatile HCN [19]. In order to avoid this drawback, the reaction of OxymaPure with carbodiimides was studied. It was found that different sterically hindered *N*,*N*′-ditertbutylcarbodiimide (DTBC) containing tertiary carbon groups did not produce either oxadiazole or HCN but showed poor reactivity. Also, the primary substituted carbodiimide EDC did not evolve into the formation of these undesirable products but showed nonproductive consumption. In this sense, a hybrid carbodiimide-based reagent, *N*,*N*′-tertbutylethylcarbodiimide (TBEC), which ensures an HCN-free reaction, was finally found to be the best reagent [20,21,22].

The problem of antimicrobial resistance is becoming a daunting challenge for human society and healthcare systems around the world. Hence, there is a constant need to develop new antibiotics to fight resistant bacteria, among other important social and economic measures. Our group has been involved in the preparation of cyclic peptide antibiotics to improve their therapeutic window and study their mechanisms of action [5,23,24,25,26,27,28,29]. The purpose of this study is to evaluate and test novel coupling reagents to optimize the synthesis of murepavadin, a novel antimicrobial peptide in clinical development, which is highly active against *Pseudomonas aeruginosa*, an infectious bacterial agent considered a serious threat to human health [30].

**Figure 2:**
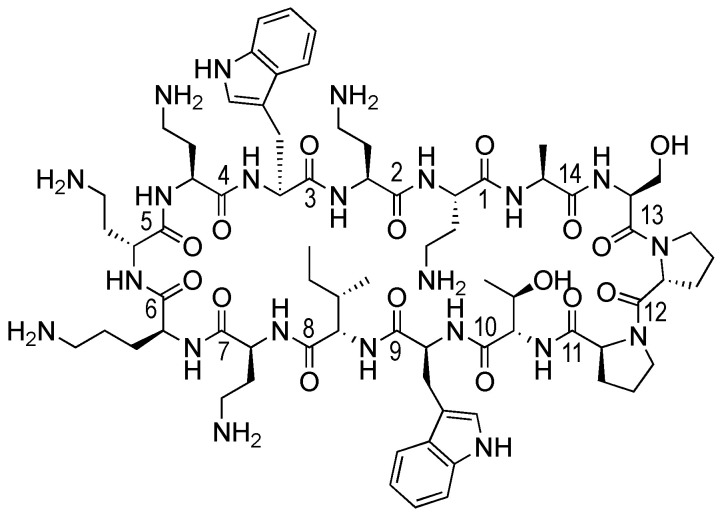
Structure of murepavadin. The sequence consists of 14 amino acids: *cyclo*[Ser-dPro-Pro-Thr-Trp-Ile-Dab-Orn-dDab-Dab-Trp-Dab-Dab-Ala].

Murepavadin (Figure 2), previously known as POL7080, is an antimicrobial peptidomimetic first-in-class of the Outer Membrane Protein Targeting Antibiotics (OMPTA), developed initially by Polyphor Ltd. (and now in the portfolio of Basilea Pharmaceutica) [31]. It is a fully synthetic non-branched cyclic peptide composed of 14 amino acids with a sequence related to that of the membranolytic host-defense peptide protegrin-I (PG-I), which contains a β-hairpin conformation stabilized by a dPro-Pro turn dipeptide [32,33]. This cyclic peptide binds to the lipopolysaccharide transport protein D (LptD), an outer membrane (OM) protein involved in the lipopolysaccharide (LPS) biogenesis in Gram-negative bacteria. It inhibits the LPS transport function of LptD, altering the OM and finally causing cell death [34,35]. Murepavadin exhibits a specific and potent bactericidal activity in vitro and in vivo against *Pseudomonas aeruginosa* [36,37,38]. Unfortunately, the phase III trials of intravenous murepavadin in hospitalized patients with pneumonia showed an unexpectedly high incidence of acute kidney injury. Currently, a clinical phase I trial evaluating an inhaled form of the antibiotic is being evaluated for the treatment of respiratory diseases, particularly cystic fibrosis and non-cystic fibrosis bronchiectasis [39,40].

## 2. Materials and Methods

### 2.1. Chemicals

2-Chlorotrityl chloride resin (2-CTC), trityl chloride resin (Cl-Trt), N-fluorenylmethoxycarbonyl (Fmoc)-protected amino acids, trifluoroacetic acid (TFA), and N-hydroxybenzotriazole (HOBt) were purchased from Fluorochem (Hadfield, UK) and Iris Biotech GmbH (Marktredwitz, Germany). *N*,*N*′-diisopropylcarbodiimide was from Thermo Fisher Scientific (Waltham, MA, USA). TBEC and Oxyma-B were given by Luxembourg Bio Technologies. Acetonitrile (ACN) HPLC gradient grade was purchased from Labkem (Barcelona, Spain). All chemicals were of the highest available purity. The solvents used were HPLC grade, except for the water, which was doubly distilled and deionized (Milli-Q system, Millipore Corp., Burlington, MA, USA).

### 2.2. Synthesis of K-Oxy-B

Oxy-B (1 g, 5.4 mmol) was dissolved in 40 mL of H_2_O. Then, 5.4 mL of KOH 1 M was slowly added until pH 7 was reached. The mixture was lyophilized, and 1.177 g of a purple/brownish solid (yield of 97%) was obtained. MS analysis: *m*/*z* = [M]^−^ = 184. HPLC analysis: purity of >99%, with relative absorption max. at 235 nm, using a Kinetex^®^ (Phenomenex, Torrance, CA, USA) reversed-phase column (4.6 × 250 mm) of a 5 μm particle diameter and a pore size of 100 Å, with 5–95% gradient of 0.036% TFA/ACN and 0.045% TFA/H_2_O over 30 min, flow = 1 mL·min^−1^ (t_R_ = 8.401 min).

### 2.3. Peptide Synthesis

Manual solid-phase peptide synthesis was performed following a Fmoc/*^t^*Bu protection strategy in polypropylene syringes fitted with a polyethylene disc, which was attached to a vacuum system for the rapid removal of solvents and any excess of reagents. 2-CTC or Cl-Trt resins (1.67 mmol·g^−1^, 250 mg) were washed with dichloromethane (DCM), DMF, and DCM (2 × 4 mL each). After resin conditioning, 2 eq of Fmoc-Dab(Boc)-OH was added along with 4 eq of DIPEA and the minimum quantity of DCM. The first coupling was left to react for 3 h at room temperature. After that, the resin was washed with DMF and DCM, capped with MeOH (0.8 mL·g^−1^resin) for 20 min, and washed again with DCM. The loading of the first amino acid residue was assessed by Fmoc quantification. The peptide sequence was assembled by the addition of the Fmoc-protected amino acid (3 eq), coupling reagent (3 eq), additive (3 eq), and the minimum quantity of DMF for 1 h. All couplings yielded ≥99%, as assessed by the Kaiser test. The Fmoc removal was achieved by successive treatments with 20% piperidine/DMF (1 × 1 min, 2 × 10 min). Cleavage of the peptide from the resin was carried out by two successive treatments with hexafluoroisopropanol (HFIP)/DCM (1:4, *v*/*v*, 3 mL·g^−1^ resin) for 1 h. The crude protected peptide was obtained after solvent evaporation under reduced pressure. For cyclization, the resulting solid was dissolved in DMF (2.5 mg·mL^−1^), and 3 eq HATU, 3 eq HOBt, and 6 eq DIPEA were added. The reaction mixture was stirred, monitored by a ninhydrin test, and after 18 h, the DMF was removed under high vacuum. To remove any excess of coupling agents, the crude product was dissolved in DCM and extracted with ACN/H_2_O (1:9, *v*/*v*). The protected peptide was isolated after solvent evaporation. The crude was treated with trifluoroacetic acid (TFA)/triisopropylsilane (TIPS)/H_2_O (95:3:2, *v*/*v*/*v*) during 90 min with stirring. Then, the reaction mixture was precipitated in ice-cold diethyl ether (70 mL), centrifuged, and washed two more times with anhydrous Et_2_O. The lyophilized peptide was dissolved in ACN/H_2_O (1:1, *v*/*v*, 100 mg·mL^−1^), purified by semipreparative HPLC, and characterized by analytical HPLC and ESI mass spectrometry.

### 2.4. Minimum Inhibitory Concentration Determination

Minimum inhibitory concentrations (MICs) of the different murepavadin batches were determined by following the Clinical and Laboratory Standards Institute (CLSI) guidelines. Bacteria used in this study were standard isolates from the American Type Culture Collection (ATCC) and German Collection of Microorganisms (DSM) (*P. aeruginosa* ATCC 27853, *P. aeruginosa* DSM 24600, and DSM 25716). Sterile microtiter plates (96 wells of 100 μL) were filled with 50 μL of cation-adjusted Mueller–Hinton broth (MHB) culture medium. Serial 2-fold dilutions of the peptides were arranged in rows ranging from 8 to 0.016 μg·mL^−1^. The last two columns were used as positive and negative controls, respectively. The bacterial suspensions prepared with an optical density at 600 nm (OD_600_) of 0.2 were diluted 100-fold, and 50 μL of the resulting suspension was added to each well (excluding the negative control). This resulted in a final concentration of approximately 5 × 10^5^ CFU·mL^−1^. The plates were incubated at 37 °C for 18–20 h, and the MICs were determined. The MIC was defined as the lowest concentration of the antimicrobial agent at which the visible growth of bacteria was prevented. Each determination was carried out in triplicate. To be considered acceptable, the three MIC results have to differ in only one well, and the result is always given as the higher of the three.

## 3. Results and Discussion

### 3.1. Murepavadin Synthesis

The synthesis of murepavadin is not described in detail in the literature. The preparation of closely related peptides (i.e., POL7001, L27-11, and L26-19) followed a general Fmoc/*^t^*Bu scheme of protection, and cyclization was performed between residues Pro^11^ (C-terminal end) and Thr^10^ (N-terminal, Figure 29) [38,41]. A different approach has been described using a native chemical ligation (NCL)/desulfurization methodology. In this approach, the cyclization site was the Dab-Ala amide bond, as an Ala amino acid is needed for this strategy. Cyclization took place in 30 min and reached a yield of 70% [42].

In our case, we decided to cyclize through the potentially less hindered peptide bond to reduce the risk of epimerization and facilitate amide formation. We avoided β-branched amino acids (Ile, Thr(*^t^*Bu)) and, in general, other *^t^*Bu- or Boc- side chain protected amino acids. The chosen bond for cyclization was the amide between Dab and Ala, obtaining yields between 89 and 96%, as described below. Hence, the first amino acid to be attached to the resin was the corresponding Fmoc-Dab(Boc)-OH (Dab^1^, as shown in Figure 2).

**Scheme 1:**
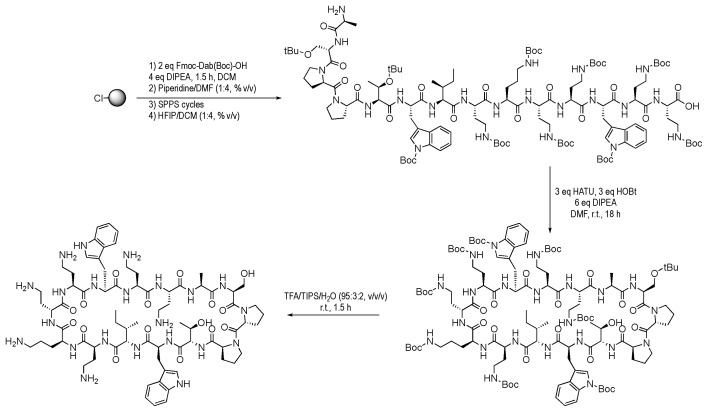
Synthesis of murepavadin.

Overall, the synthesis of murepavadin was undertaken by SPPS following a Fmoc/*^t^*Bu scheme of protection on the trityl-type of resins using several acylating agents, as indicated in Table 1 [38]. After the assembly of the linear protected peptide sequence on resin, a mild acidolysis using HFIP/DCM (1:4) was performed in order to obtain the partially protected peptide. The cyclization of the lineal crude was achieved with 3 eq *O*-(7-azabenzotriazol-1-yl)-1,1,3,3-tetramethyl-uronium hexafluorophosphate (HATU), 3 eq HOBt, and 6 eq *N*,*N*-diisopropylethylamine (DIPEA). After cyclization, the crude protected peptide was treated with an acidolysis reagent composed of TFA/TIPS/H_2_O (95:3:2, *v*/*v*/*v*) to obtain the totally deprotected cyclic peptide (Scheme 1). The purity of the fully deprotected peptide was assessed by HPLC by the integration of peaks at 220 nm.

**Figure 3:**
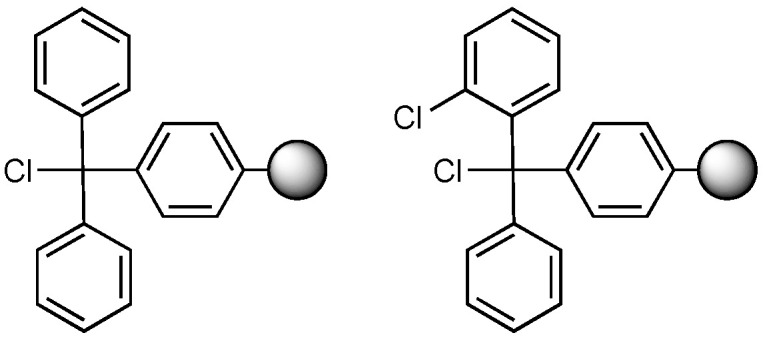
Trityl-based polystyrene solid supports: trityl chloride resin (**left**), 2-chlorotrityl chloride resin (**right**).

Two different trityl-based solid supports (2-CTC and Cl-Trt) were evaluated (Figure 3). In the conditions used, we found that the synthesis performed using 2-CTC resin (Table 1, synthesis A) yielded higher loading in comparison to Cl-Trt (Table 1, synthesis C). Dealing with highly functionalized resins can sometimes be disadvantageous, especially when treating with difficult peptide sequences, as they can lead to uncomplete acylation and make re-coupling of the amino acids necessary [43,44]. However, in the present case, the assembly was quite straightforward, and only a few re-couplings were needed in some of the syntheses performed. According to the results, 2-CTC seems to be a better option than Cl-Trt in terms of loading and overall yield (Table 1 and Table 2). Therefore, 2-CTC was the resin of choice in this study.

When HOBt was used as the additive in carbodiimide (DIC) acylations, a low resin detachment yield was observed in both cases (entries A and C, Table 1). This result could be expected according to the literature since the use of HOBt has been described to cause premature peptide cleavage during assembly on 2-CTC resin due to its acidic character [13]. Similarly, OxymaPure (Luxembourg Bio Technologies, Ness Ziona, Israel) has also been reported to cause the same problem of peptide losses. For this reason, we decided to perform the synthesis using K-Oxyma (a conjugated base of OxymaPure) instead of HOBt or OxymaPure [18]. The resin detachment yield increased from 46 to 91% on the 2-CTC resin (entry B).

We then decided to test the novel protocol involving TBEC. Originally, the TBEC protocol was developed to avoid the formation of oxadiazole and HCN due to the reaction of DIC and OxymaPure [21,22]. In order to avoid losses during the assembly, we used TBEC in combination with K-Oxyma and the newly described additives Oxy-B and its conjugate base K-Oxy-B [18,21,45,46]. According to the literature, the use of K-Oxyma prevents premature peptide detachment from 2-chlorotrityl resin. Our results show that both Oxy-B and K-Oxy-B additives performed satisfactorily from this point of view. All syntheses rendered comparable detachment yields, ranging from 89 to 93% (entries D to F), and much better than the HOBt-based assemblies and similar to DIC/K-Oxyma. This fact could be attributed to the lower acidic character of Oxy-B (pK_a_ 8.20) in comparison with the higher acidity of HOBt and OxymaPure (the pKa for both is 4.60) [20]. Finally, it can be seen that both Oxy-B and K-Oxy-B performed similarly well. Therefore, there is no apparent need to prepare the corresponding conjugate base of Oxy-B.

In all syntheses, after the acidolysis of the peptidyl resins, the linear protected peptide was cyclized. Macrolactamization was performed in solution using HATU, HOBt, and DIPEA (3:3:6 mole ratio) according to the literature [38]. The cyclization yields ranged from 89 to 96%. It is worth mentioning that this reaction was monitored by ninhydrin since both the linear and cyclic protected peptides did not elute by HPLC using ACN/H_2_O mixtures.

Total deprotection was achieved by treatment with TFA/TIPS/H_2_O (95:3:2, *v*/*v*/*v*%) to obtain the cyclic deprotected peptide. After precipitation in anhydrous diethyl ether and isolation of the crude peptides, the yields ranged from 83 to 91%.

**Figure 4:**
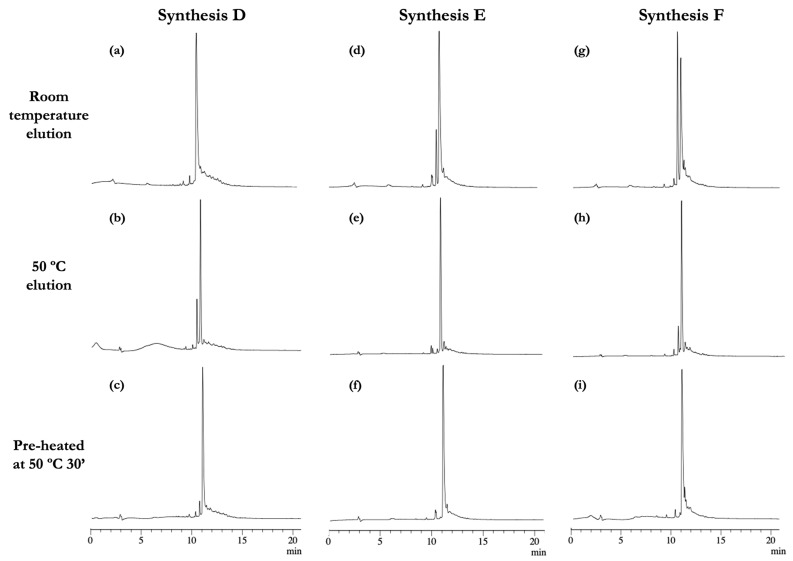
Chromatograms of murepavadin crudes obtained after treating the protected peptide crude with TFA/TIPS/H_2_O (95:3:2, *v*/*v*/*v*) using different chromatographic conditions: (**a**) Synthesis D, HPLC 25 °C, (**b**) Synthesis D, HPLC column *T* set to 50 °C, (**c**) Synthesis D, sample pre-heating 50 °C, (**d**) Synthesis E, HPLC 25 °C, (**e**) Synthesis E, HPLC column *T* set to 50 °C, (**f**) Synthesis E, sample pre-heating 50 °C, (**g**) Synthesis F, HPLC 25 °C, (**h**) Synthesis F, HPLC column *T* set to 50 °C, (**i**) Synthesis F, sample pre-heating 50 °C.

The assessment of the purity of the different batches was not a straightforward task. As mentioned before, murepavadin contains a dPro-Pro moiety within the sequence. It is well known that proline residues may lead to conformational equilibria due to the possibility of significant populations of both the *cis* and *trans* conformers [47,48]. When assessing purity, we sometimes found out that two peaks with the same *m*/*z* ratio were obtained by HPLC-MS (Figure 4d,g). However, if the samples (previously lyophilized) were pre-heated to 50 °C for 30 min or the chromatography was performed using an oven at 50 °C, the coalescence of peaks took place into a single major peak (Figure 4e,f,h). In all cases, the purity was determined by the integration of peaks at 220 nm corresponding to a *m*/*z* ratio of a 777.9 mass unit, which corresponds to the [M+2H^+^]^+2^ ion, as determined by ESI-MS.

The final overall crude yields ranged from 70 to 80% in all cases except those involving the use of HOBt (30–40%). Crude purities went from 58 to 67%. Hence, the use of K-Oxyma, Oxy-B, and K-Oxy-B yielded similar good results. The highest purity (67%) was obtained for the combination of TBEC/Oxy-B in the 2-CTC resin, as shown in Table 1 and Figure 4.

After purification by semipreparative HPLC, all the murepavadin batches were obtained with a purity higher than 99%. As seen in Table 2, global yields of synthesis A and C were clearly affected by premature loss during the peptide assembly due to the acidic nature of HOBt. In comparison, the syntheses performed using K-Oxyma, Oxy-B, and K-Oxy-B rendered much higher yields (Table 2 and Syntheses B, D, E, and F). As expected, the higher yields and purities obtained for the crudes were reflected in the global yield after purification. Oxy-B is as effective as its conjugate base; hence, there is no need to prepare the potassium salt of Oxy-B (K-Oxy-B) in clear contrast to the pair OxymaPure/K-Oxyma described in the literature. In our hands, the best yield for the total synthesis of murepavadin in the 2-CTC resin was obtained for the acylating agents TBEC/Oxy-B.

### 3.2. Antimicrobial Activity Determination of the Different Murepavadin Batches

As mentioned before, murepavadin exhibits potent activity against *P. aeruginosa* by targeting the periplasmatic β-jellyroll domain of the LptD protein of this bacteria. It is well known that drug–protein interactions are stereospecific, and studies have shown that murepavadin exerts its antipseudomonal activity by means of a mechanism of action involving a chiral receptor [49,50]. In this sense, we wanted to confirm the chiral integrity of the murepavadin purified batches by assessing their in vitro antibacterial activity. Antimicrobial activity was determined in terms of minimal inhibitory concentrations (MICs) by the broth dilution method and following the CLSI guidelines.

All murepavadin batches were tested against three different *P. aeruginosa* strains. Two of them (DSM 24600 [51] and DSM 25716 [52]) were resistant to ß-lactam antibiotics. *P. aeruginosa* DSM 24600 produces extended-spectrum ß-lactamase (ESBL) and is carbapenem-resistant (Genotype blaVIM-1). *P. aeruginosa* DSM 25716 is resistant to carbapenems (i.e., imipenem) as it expresses a VIM ß-lactamase but it does not produce extended-spectrum ß-lactamases (ESBL).

As seen in Table 3, this cyclic peptide exerts a potent antipseudomonal activity, with *P. aeruginosa* DSM 24600 being the most susceptible strain. The MIC values are in the range of those found in the literature (0.008–0.25 μg·mL^−1^) [36,38]. No differences were observed between the different batches, which indicates that the chiral integrity of the molecule was preserved in the different syntheses of murepavadin.

## 4. Conclusions

The preparation of murepavadin, a complex macrocyclic peptide antibiotic, was successfully achieved. Its assembly was accomplished using different resins and coupling reagents. Regarding the resin, 2-CTC was preferred over Cl-Trt, as the loading obtained with this solid support was higher, and the peptide assembly was quite straightforward. When using HOBt in combination with the standard carbodiimide DIC for peptide assembly, low detachment yields were obtained (41–46%), indicating premature cleavage of the peptide chains, probably due to the acidic nature of this additive. The cleavage yield was improved (91%) when the conjugate base of OxymaPure, K-Oxyma, was used with DIC. Also, different oxyma-based additives were used in combination with a relatively new carbodiimide, TBEC, and proved to be as effective as the system DIC/K-Oxyma in terms of cleavage yield (89–93%). The use of K-Oxyma/DIC, Oxy-B/TBEC, and K-Oxy-B/TBEC yielded similar good yields and purities. Oxy-B is as effective as its conjugate base; hence, there is no need to prepare the potassium salt of Oxy-B (K-Oxy-B) in clear contrast to the pair OxymaPure/K-Oxyma. Altogether, the best yield for the total synthesis of murepavadin was obtained using the 2-CTC resin and the acylating agents TBEC/Oxy-B. Chromatographic elution by HPLC at a high temperature (50 °C or pre-heating samples at 50 °C) was necessary for the correct assessment of murepavadin crude purity.

Finally, the antimicrobial activity of the different synthetic batches was tested in three *P. aeruginosa* strains, including highly resistant varieties that consisted of two carbapenem-resistant strains and an ESBL producer. All murepavadin batches yielded the same MIC values, which demonstrates that the chiral integrity of the molecule was preserved throughout the whole synthetic procedure.

## Figures and Tables

**Table 1:** Yields and purities for each of the syntheses performed (also see Table S1).

Synthesis	Resin	Loading	Coupling Agents	Resin Detachment Yield	Cyclization Yield	Acidolysis Yield	Overall Crude Yield	Crude Purity ^a^
A	2-CTC	0.86 mmol/g	3 eq DIC 3 eq HOBt	46%	96%	90%	40%	58%
B	2-CTC	0.86 mmol/g	3 eq DIC 3 eq K-Oxyma	91%	93%	83%	70%	64%
C	Cl-Trt	0.45 mmol/g	3 eq DIC 3 eq HOBt	41%	89%	83%	30%	67%
D	2-CTC	0.63 mmol/g	3 eq TBEC 3 eq K-Oxyma	90%	95%	88%	75%	60%
E	2-CTC	0.69 mmol/g	3 eq TBEC 3 eq Oxy-B	89%	96%	89%	76%	67%
F	2-CTC	0.70 mmol/g	3 eq TBEC 3 eq K-Oxy-B	93%	94%	91%	80%	59%

^a^ The HPLC purities were obtained by integrating the peak areas at 220 nm.

**Table 2:** Purification and global yields for each of the syntheses performed.

Synthesis	Resin	Coupling Agents	Purification Yield	Global Yield
A	2-CTC	3 eq DIC 3 eq HOBt	29%	12%
B	2-CTC	3 eq DIC 3 eq K-Oxyma	40%	28%
C	Cl-Trt	3 eq DIC 3 eq HOBt	45%	14%
D	2-CTC	3 eq TBEC 3 eq K-Oxyma	36%	27%
E	2-CTC	3 eq TBEC 3 eq Oxy-B	39%	30%
F	2-CTC	3 eq TBEC 3 eq K-Oxy-B	34%	27%

**Table 3:** MIC values (μg·mL^−1^) for the different batches of murepavadin.

	A	B	C	D	E	F
*P. aeruginosa* ATCC 27853	0.0625	0.0625	0.0625	0.0625	0.0625	0.0625
*P. aeruginosa* DSM 24600	0.03125	0.03125	0.03125	0.03125	0.03125	0.03125
*P. aeruginosa* DSM 25716	0.25	0.25	0.25	0.25	0.25	0.25

## Data Availability

Dara are contained within the article and Supplementary Materials.

## Supplementary Materials

The following supporting information can be downloaded at: https://www.mdpi.com/article/10.3390/biom14050526/s1, Table S1: Quantities obtained in each step of the synthesis of murepavadin for the different batches; Figure S1: HPLC and MS analysis of K-Oxy-B; Figures S2–S7: HPLC and MS analysis of murepavadin batches A to F.
