# Supplementary material for: Synthesis of the Antimicrobial Peptide Murepavadin Using Novel Coupling Agents
Source: Biomolecules. 2024 Apr 27;14(5):526. doi: 10.3390/biom14050526 (PMC11117477; doi:10.3390/biom14050526)
Supplement: Supplementary file 1 [file biomolecules-14-00526-s001.zip › biomolecules-2975195-supplementary.pdf]

Supporting information

Table S1. Summary of yields and purities of the different synthetic batches of murepavadin

Entry	Resin	Initial resin amount / loading	Coupling agents	Cleavage yield	Cyclization yield	Acidolysis yield	Crude purity	Purification yield	Global yield
**A**	2-CTC	253.9 mg<br>0.86 mmol/g	3 eq DIC<br>3 eq HOBt	249.1 mg<br>46 %	239.1 mg<br>96 %	194.5 mg<br>90 %	58 %	56.1 mg<br>29 %	12 %
**B**	2-CTC	264.4 mg<br>0.86 mmol/g	3 eq DIC<br>3 eq K-Oxyma	513.0 mg<br>91 %	476.0 mg<br>93 %	358.4 mg<br>83 %	64 %	143.4 mg<br>40 %	28 %
**C**	Cl-Trt	258.4 mg<br>0.45 mmol/g	3 eq DIC<br>3 eq HOBt	118.9 mg<br>41 %	104.9 mg<br>89 %	78.9 mg<br>83 %	67 %	35.2 mg<br>45 %	14 %
**D**	2-CTC	245.5 mg<br>0.63 mmol/g	3 eq TBEC<br>3 eq K-Oxyma	344.8 mg<br>90 %	325.0 mg<br>95 %	260.0 mg<br>88 %	60 %	93.2 mg<br>36 %	27 %
**E**	2-CTC	256.4 mg<br>0.69 mmol/g	3 eq TBEC<br>3 eq Oxy-B	391.2 mg<br>89 %	372.9 mg<br>96 %	301.4 mg<br>89 %	67 %	117.5 mg<br>39 %	30 %
**F**	2-CTC	258.1 mg<br>0.70 mmol/g	3 eq TBEC<br>3 eq K-Oxy-B	415.8 mg<br>93 %	389.0 mg<br>94 %	319.8 mg<br>91 %	59 %	108.7 mg<br>34 %	27 %

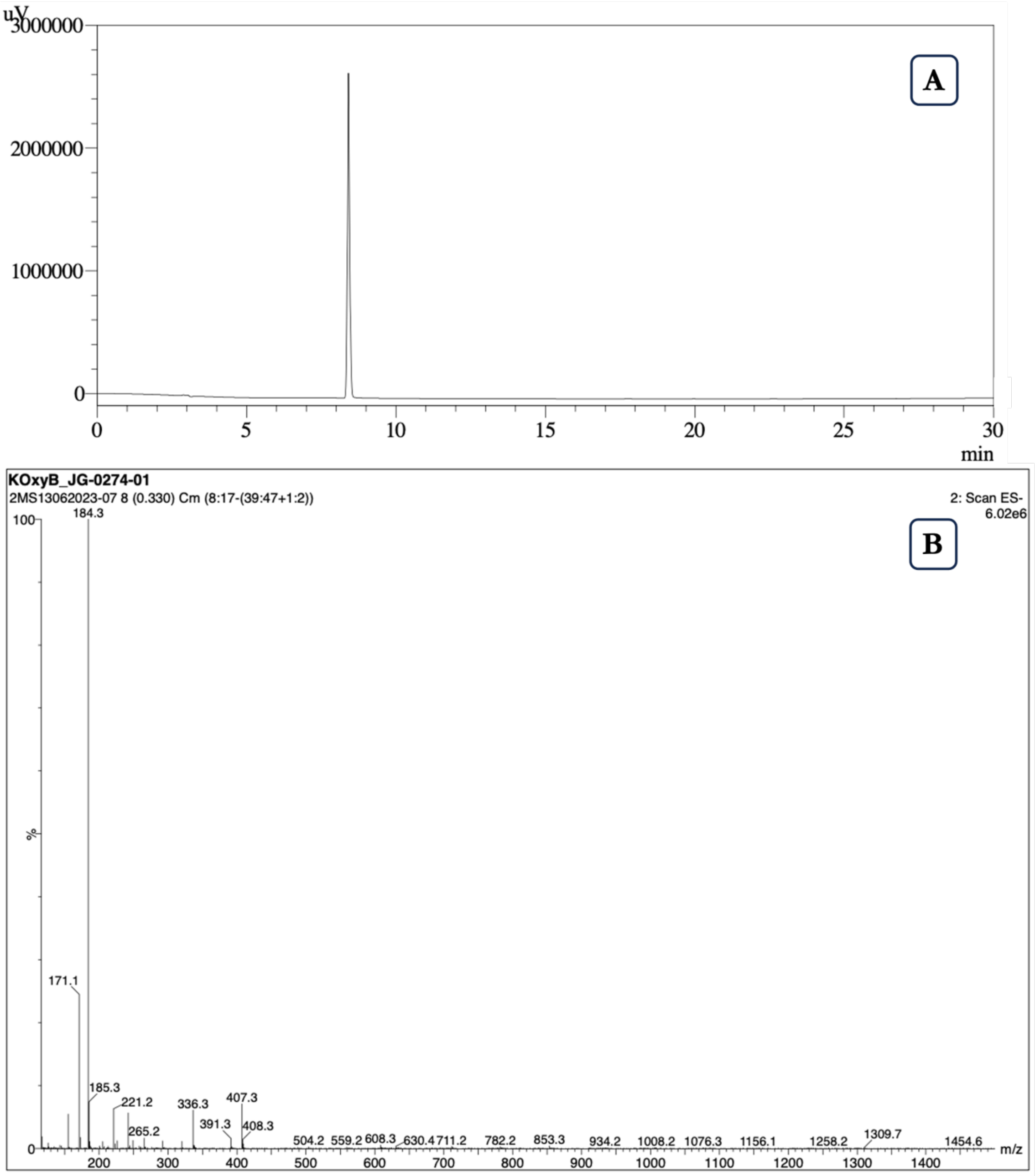

Figure S1. HPLC (A) and ESI-MS (B) analysis of K-Oxy-B.

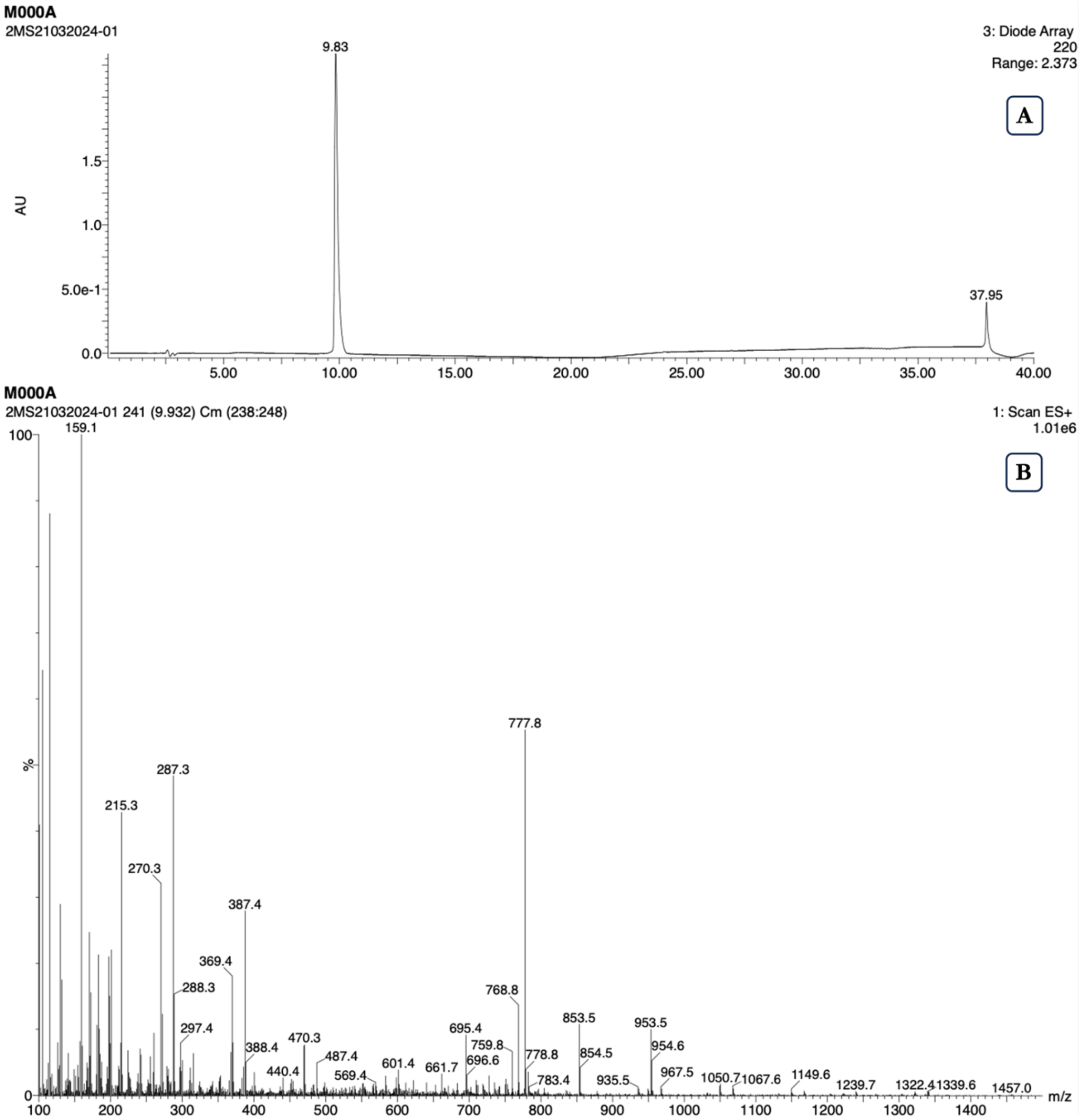

Figure S2. HPLC (A) and ESI-MS (B) analysis of murepavadin batch A.

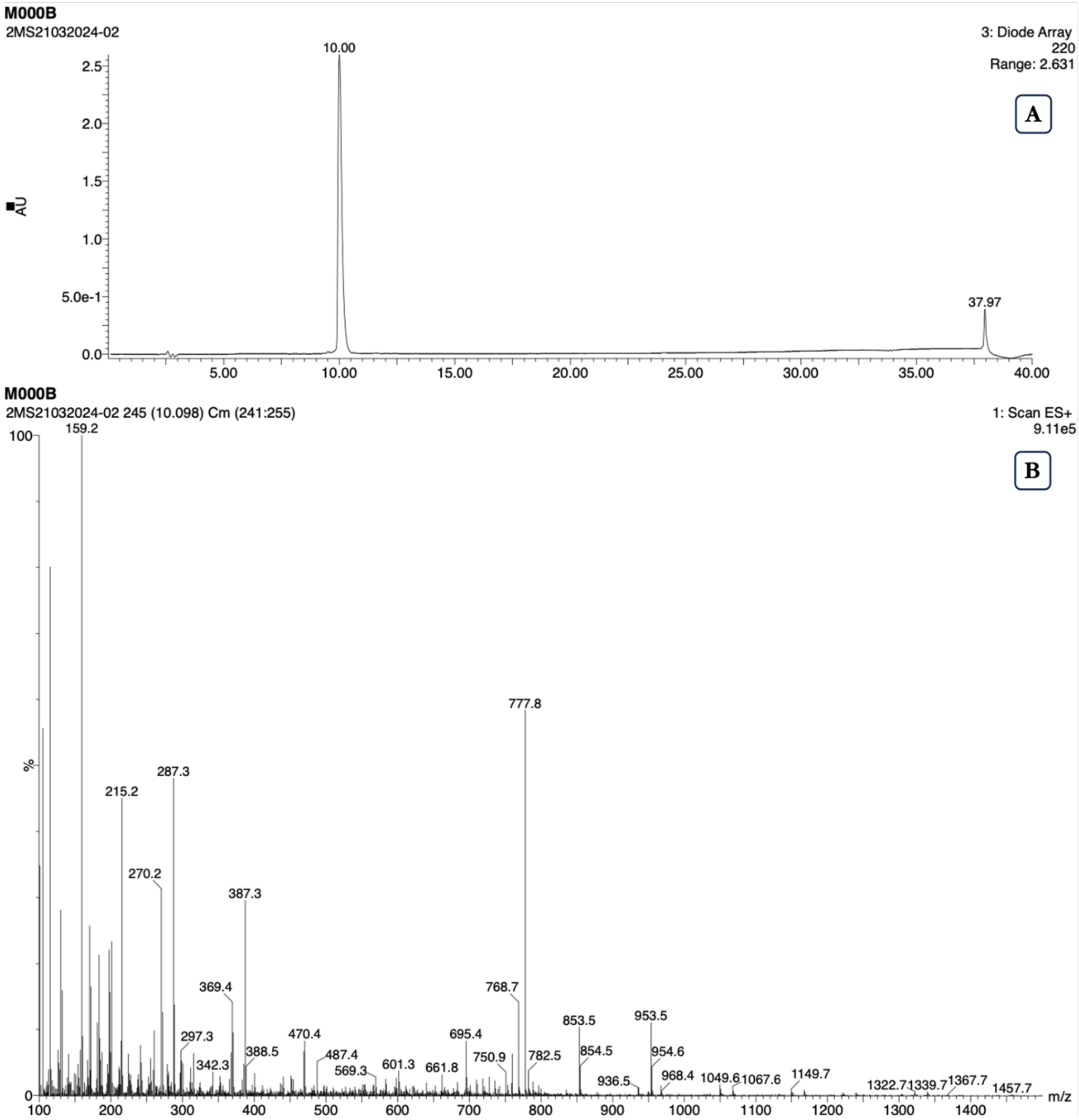

Figure S3. HPLC (A) and ESI-MS (B) analysis of murepavadin batch B.

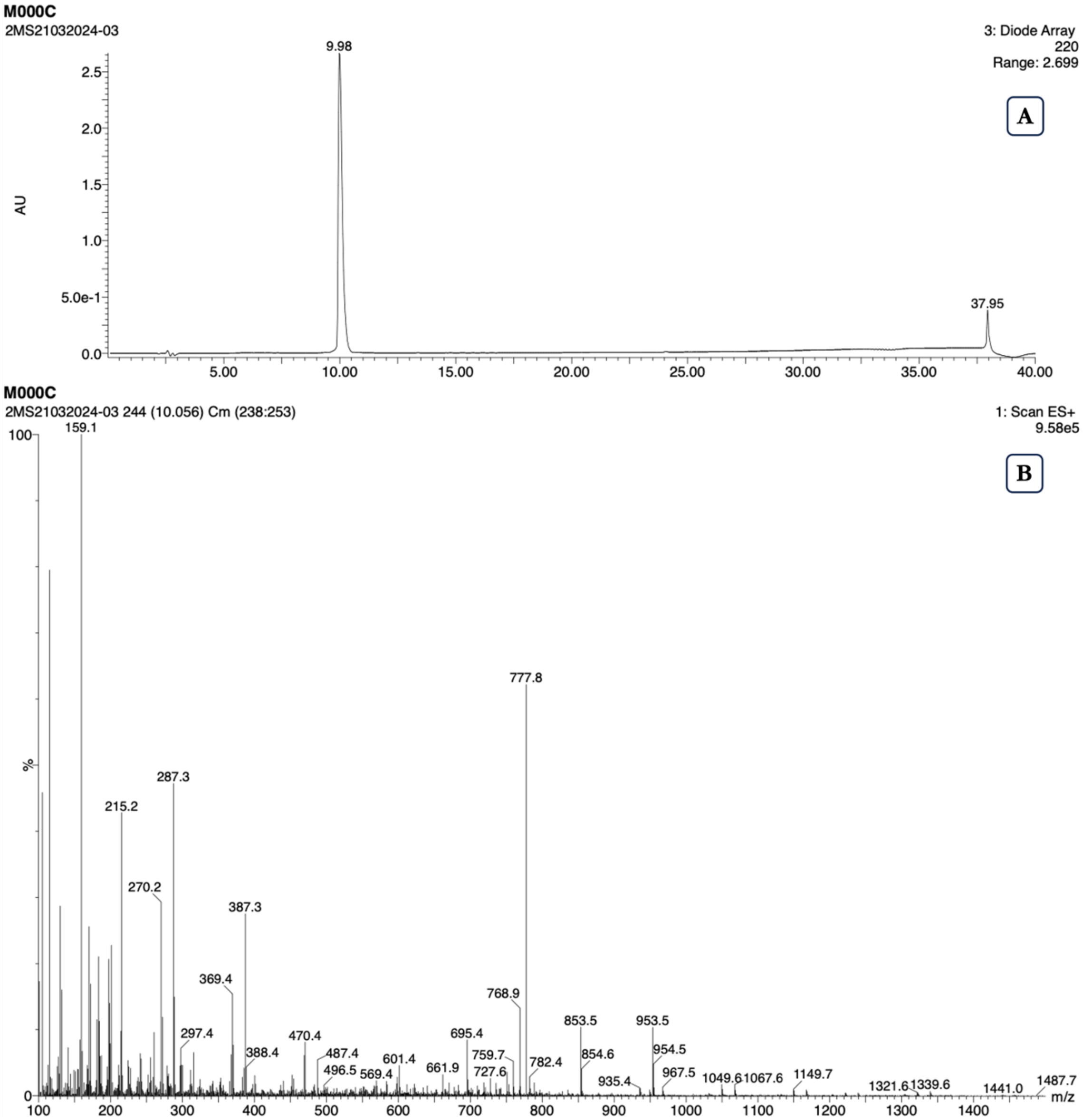

Figure S4. HPLC (A) and ESI-MS (B) analysis of murepavadin batch C.

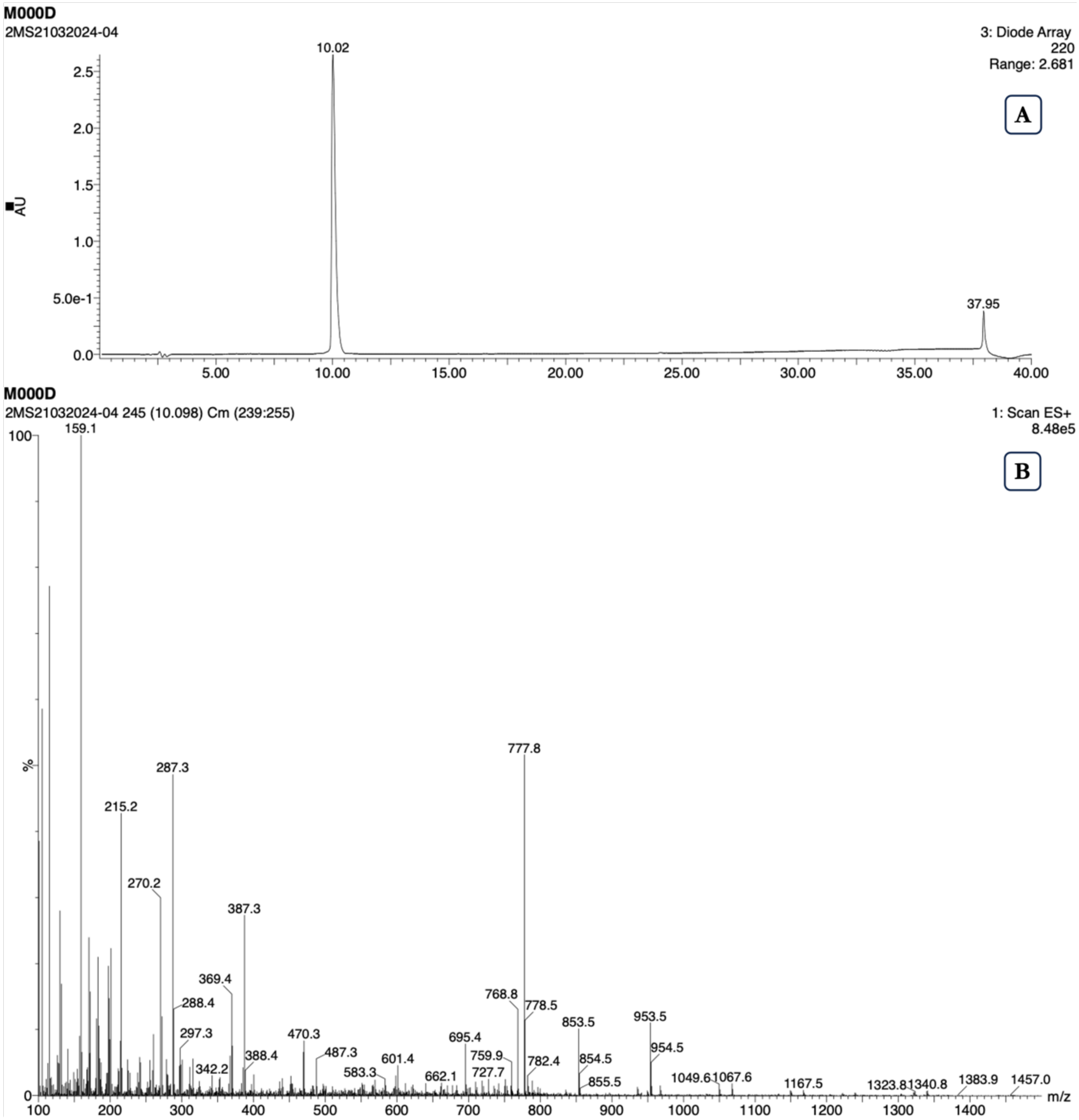

Figure S5. HPLC (A) and ESI-MS (B) analysis of murepavadin batch D.

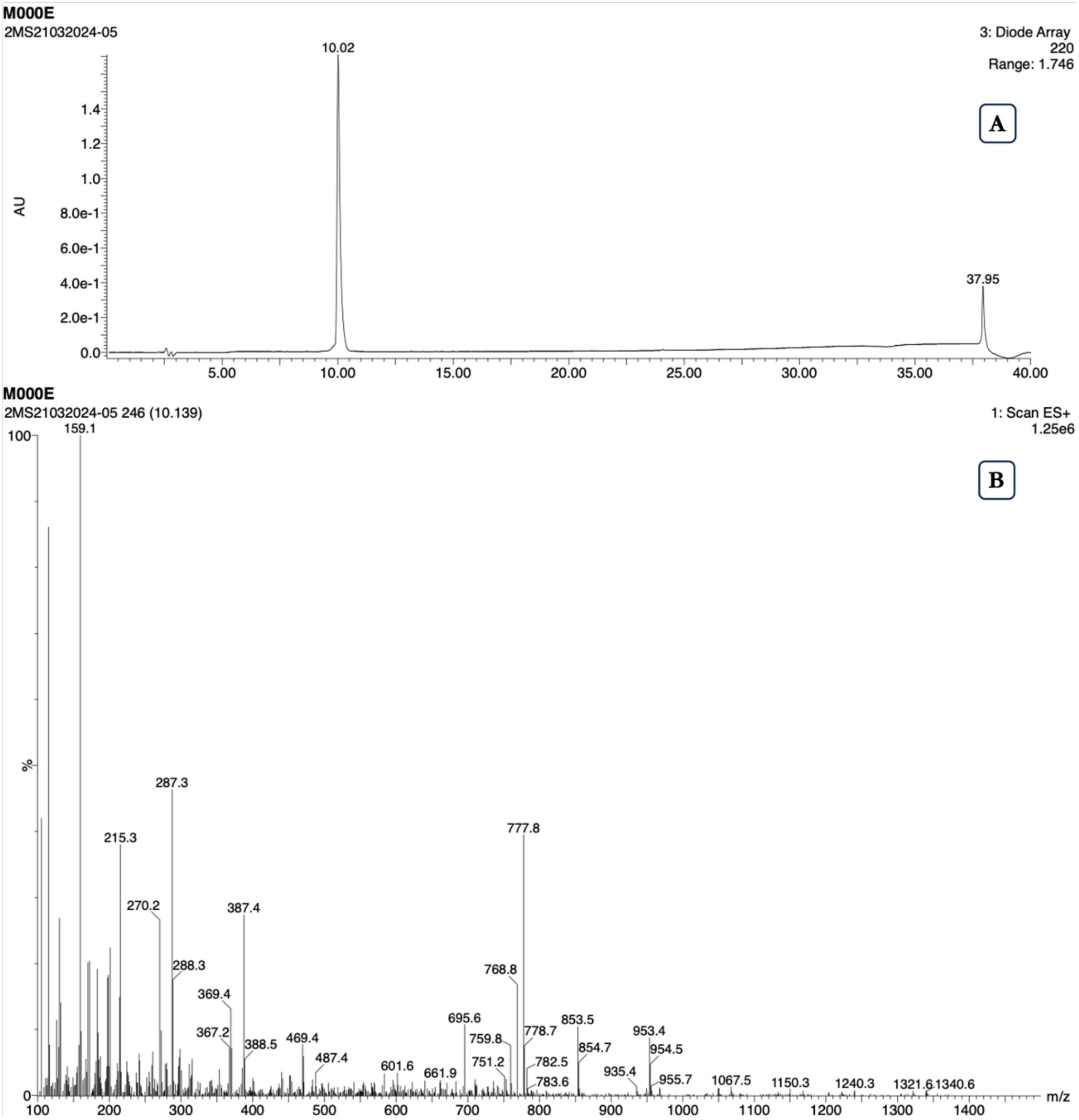

Figure S6. HPLC (A) and ESI-MS (B) analysis of murepavadin batch E.

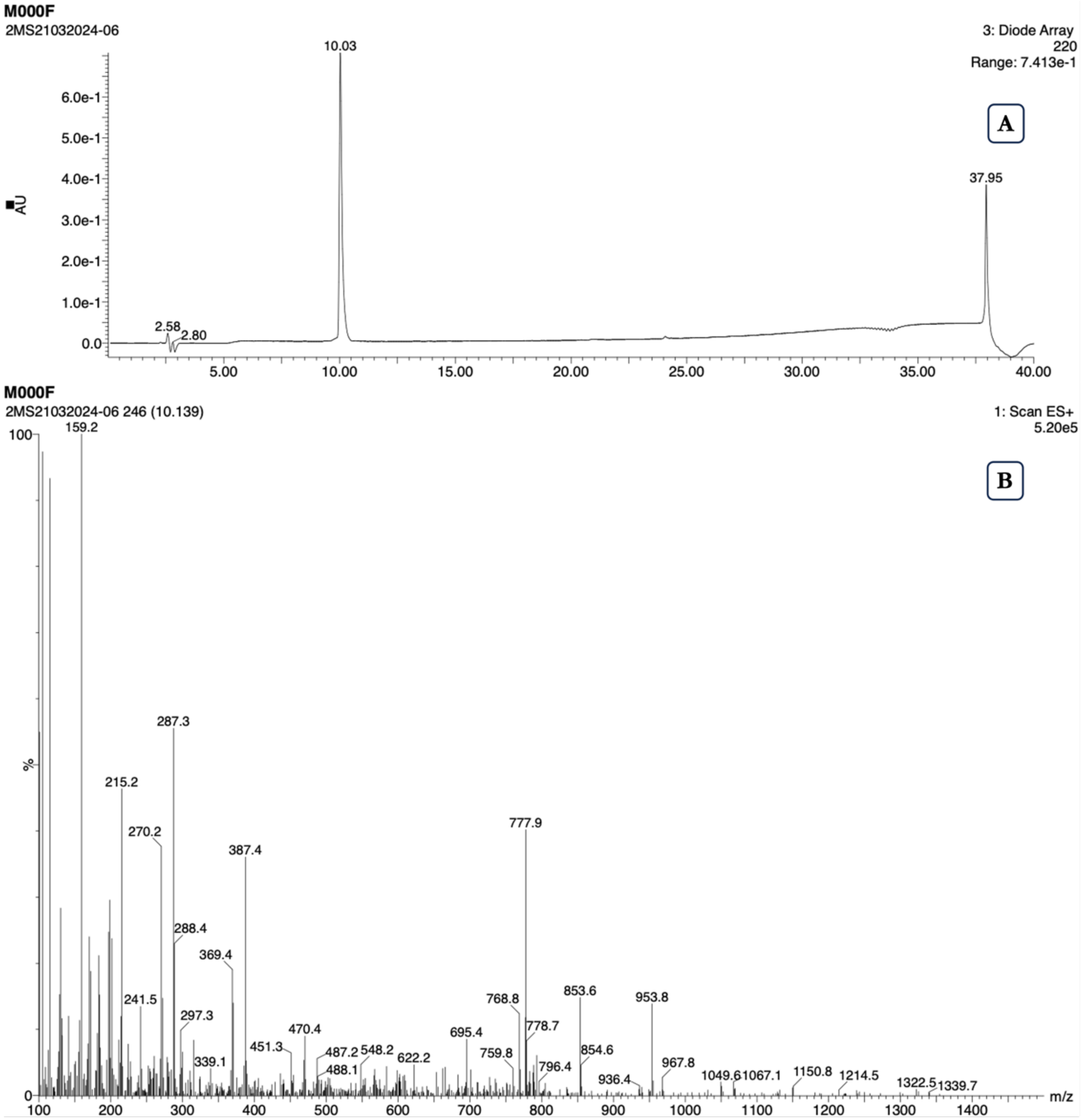

Figure S7. HPLC (A) and ESI-MS (B) analysis of murepavadin batch F.
